# Endoscopic and histopathologic features of Anti‐PD‐1‐related collagenous colitis

**DOI:** 10.1002/deo2.92

**Published:** 2022-01-11

**Authors:** Hidezumi Kikuchi, Hirotake Sakuraba, Yui Akemoto, Kazuhiro Hosoi, Yasuhisa Murai, Kentaro Hoshi, Yukari Fukutoku, Taka Asari, Yohei Sawada, Keisuke Hasui, Tetsuya Tatsuta, Hiroto Hiraga, Daisuke Chinda, Tatsuya Mikami, Shinsaku Fukuda

**Affiliations:** ^1^ Department of Gastroenterology and Hematology Hirosaki University Graduate School of Medicine Aomori Japan; ^2^ Department of Community Medicine Hirosaki University Graduate School of Medicine Aomori Japan; ^3^ Department of Anatomic Pathology Hirosaki University Hospital Aomori Japan; ^4^ Department of Pharmacy Hirosaki University Hospital Aomori Japan; ^5^ Innovation Center for Health Promotion Hirosaki University Graduate School of Medicine Aomori Japan

**Keywords:** CD8‐positive T‐lymphocytes, collagenous colitis, diarrhea, immune checkpoint inhibitor, proton pump inhibitor

## Abstract

**Objectives:**

Cancer patients treated with immune checkpoint inhibitors occasionally show persistent diarrhea accompanied by endoscopic features of ulcerative colitis. The endoscopic mucosal inflammation may appear mild in some patients compared to the clinical severity, which can make choosing a treatment challenging. In this study, we evaluated the factors that support the continuation of chemotherapy by assessing the endoscopic and histopathological characteristics of patients who experienced diarrhea after immune checkpoint inhibitor administration.

**Methods:**

This study included eight patients who were diagnosed with collagenous colitis based on pathological assessments. We retrospectively investigated these patients’ backgrounds, laboratory data, and computed tomography images that were extracted from their medical records. We also summarized their endoscopic and pathologic findings.

**Results:**

All eight patients were being treated with anti‐programmed cell death‐1/programmed cell death‐ligand 1 therapeutic agents and had a recent history of oral proton pump inhibitor therapy. The anti‐programmed cell death‐1‐related collagenous colitis in these cases was characterized by endoscopically mild mucosal inflammation, high fecal calprotectin levels, and a lower frequency of intestinal wall thickening on computed tomography. Histological assessments showed CD8^+^ lymphocytes predominantly infiltrating the lamina propria and crypts of the colonic mucosa. Suspending the proton pump inhibitor therapy relieved the patients’ symptoms and allowed the continuation of the anti‐programmed cell death‐1/programmed cell death‐ligand 1 therapy.

**Conclusions:**

Anti‐programmed cell death‐1‐related collagenous colitis is reversible; appropriate diagnosis of adverse events is crucial for the continuation of immune checkpoint inhibitor therapy.

## INTRODUCTION

Immune checkpoint inhibitors (ICIs) are approved for the treatment of various types of cancers; however, immune‐related adverse events (irAEs) are a major problem associated with ICI treatments.^1^ Colitis is a common gastrointestinal irAE,^2^, ^3^ and although the pathogenic mechanisms underlying colitis presenting as an irAE remain unknown, the endoscopic features resemble those of ulcerative colitis.^4^, ^5^, ^6^, ^7^ There are two types of irAE colitis: active and lymphocytic.^2^, ^3^, ^8^ Symptomatic therapy or suspension of ICIs is effective for treating mild colitis manifesting as an irAE; however, severe cases require administration of steroids or biologics, such as infliximab.^5^ Early intervention can be key to enable continued chemotherapy for patients with colitis. Unfortunately, creating a medical plan for ICI‐related diarrhea with poor endoscopic findings may be difficult.

A few reports have described collagenous colitis (CC) development after ICI treatments; however, the underlying mechanisms and clinical features remain unclear.^9^, ^10^, ^11^ irAEs resulting from an ICI treatment can systemically affect several organs, and thus, involvement of a single inflammatory mechanism may be unlikely.^12^ The intestinal immune system crosstalk is particularly complex and controversial. Therefore, the etiology of colitis manifesting as an irAE has not yet been fully clarified. In this study, we collected data from patients who developed CC after undergoing anti‐programmed cell death‐1 (anti‐PD‐1)/programmed cell death‐ligand 1 (PD‐L1) treatments at a single institution and examined the endoscopic and pathological characteristics associated with anti‐PD‐1‐related CC.

## METHODS

### Patients

Endoscopic imaging and histopathological evaluations of colonic mucosal biopsies were performed from January 2016 to December 2020 for every patient who visited our department presenting with diarrhea after undergoing ICI treatment. We included patients diagnosed with CC in this study; patients’ clinical information related to age, sex, medical history, diarrhea severity, type of ICI, period from ICI administration to symptom onset, and concomitant medications was retrospectively extracted from the medical records. Diarrhea severity was graded using the Common Terminology Criteria for Adverse Events version 5.0 (National Cancer Institute/National Institute of Health, Bethesda, MD, USA).^13^ Laboratory data, including C‐reactive protein (CRP) and fecal calprotectin (FC) levels and results of fecal immunochemical test (FIT), were obtained, and contrast‐enhanced computed tomography (CT) images during the symptomatic diarrhea period were acquired before patient colonoscopy.

Additionally, to compare CC features with or without ICI treatment, data for nine patients with ICI‐independent traditional CC (T‐CC) were extracted from a pathology database at our institution.

### Colonoscopy

A colonoscopy was performed for each patient within 4 days of their first visit to our department. Loss of vascularity, erythema, granularity, erosion, and ulcers was defined as endoscopic mucosal changes. Biopsies from each colon segment (i.e., cecum, ascending colon, transverse colon, descending colon, and sigmoid colon) and the rectum were performed for six patients. The colonoscope was inserted into the transverse colon of one patient due to poor bowel preparation; subsequently, biopsy specimens were collected from the transverse colon to the rectum. Biopsy specimens could not be collected from the cecum and ascending colon of another patient who had undergone a right hemicolectomy.

### Pathological evaluation

CC was defined by a subepithelial collagen band thickness >10 μm using Masson's trichrome staining. A tissue architecture assessment using hematoxylin and eosin staining and simple mucosal biopsy criteria was required to exclude inflammatory bowel disease (IBD).^14^ We investigated T‐lymphocyte infiltration into the colorectal mucosa using immunohistochemistry with the following antibodies: CD3 (prediluted; Leica Biosystems, Wetzlar, Germany), CD4 (1:4 dilution; Nichirei Corporation, Tokyo, Japan), and CD8 (1:150 dilution; Nichirei Corporation, Tokyo, Japan). We enumerated CD3^−^, CD4^−^, and CD8‐positive T cells per 200× magnification field‐of‐view.

### Statistical analysis

Statistical analyses were performed using a Mann–Whitney test (GraphPad Prism 9.0.2; GraphPad Software, San Diego, CA, USA).

## RESULTS

### Clinical characteristics

During the study period, 18 patients visited our department reporting diarrhea after ICI administration; eight were diagnosed with CC based on subepithelial collagen band deposition >10 μm. The differential diagnoses for these patients were made based on the exclusion of other types of infectious colitis through fecal bacterial culture, *Clostridium difficile* toxin test, and by the absence of endoscopic and histological findings for cytomegalovirus or tuberculosis infections. The remaining 10 patients were diagnosed with irAE colitis (n = 4), irAE ileitis (n = 1), *C. difficile* infection (n = 1), eosinophilic colitis (n = 1), nonspecific colitis (n = 1), and nonspecific ileitis (n = 2). The irAE colitis diagnoses were based on histopathological colitis patterns, characterized by neutrophilic inflammation and prominent intestinal epithelial cell apoptosis, in the presence of anti‐PD‐1 therapy.^15^ None of the patients met the diagnostic criteria for lymphocytic colitis or IBD.

The clinical characteristics of the patients are summarized in Table 1. There were five men and three women with a median age of 76 years (range, 57–83 years) and a variety of oncological backgrounds. All the patients received anti‐PD‐1/PD‐L1 antibody therapy. Seven patients underwent ICI monotherapy, with nivolumab (n = 4), pembrolizumab (n = 2), or durvalumab (n = 1). One patient underwent nivolumab plus ipilimumab combination therapy. None of the patients had hematochezia. Surprisingly, all the patients were taking oral proton pump inhibitors (PPIs), including lansoprazole (n = 6), and esomeprazole (n = 2). Four patients also received analgesic drugs, non‐steroidal anti‐inflammatory drugs (naproxen), acetaminophen, or codeine phosphate.

**TABLE 1:** Patient characteristics

						ICI		Concomitant drugs		
Case	Age	Sex	Original cancer	Symptom	CTCAE (grade)	Drug	Time between start of ICI and diarrhea (weeks)	PPI and suspicious drugs	Time between start of drugs and diarrhea (weeks)	Other drugs
1	78	F	NSCLC	Diarrhea	2	Niv	59	Lansoprazole	201	Olanzapine, zolpidem, magnesium oxide
2	57	M	Esophageal cancer	Diarrhea	1	Niv	109	Lansoprazole	117	Bromhexine hydrochloride, Hochuekkito
3	65	M	NSCLC	Diarrhea	2	Dur	5	Lansoprazole acetaminophen	13 13	Clonazepam, tapentadol hydrochloride, pregabalin, codeine phosphate
4	83	M	Renal cell carcinoma	Diarrhea	2	Niv+Ipi	3	Esomeprazole naproxen	4 2	Nifedipine, valsartan, brotizolam, glimepiride amlodipine besilate,
5	83	F	Urothelial carcinoma	Diarrhea	3	Pem	23	Esomeprazole	35	Atorvastatin, nifedipine, carbazochrome sodium sulfonate hydrate
6	74	M	NSCLC	Diarrhea	2	Niv	16	Lansoprazole acetaminophen	60 4	Allopurinol, dutasteride, silodosin, tapentadol hydrochloride
7	71	M	NSCLC	Diarrhea	2	Pem	13	Lansoprazole	17	Fexofenadine hydrochloride
8	82	F	Malignant lymphoma	Diarrhea	2	Niv	6	Lansoprazole naproxen	11 6	Edoxaban tosilate hydrate, bisoprorol

Abbreviations: PPI, proton pump inhibitor, CTCAE, Common Terminology Criteria for Adverse Events; NSCLC, non‐small cell lung cancer; ICI, immune checkpoint inhibitor; Niv, nivolumab; Dur, durvalumab; Ipi, ipilimumab; Pem, pembrolizumab.

### Endoscopic and histological findings

**FIGURE 1:**
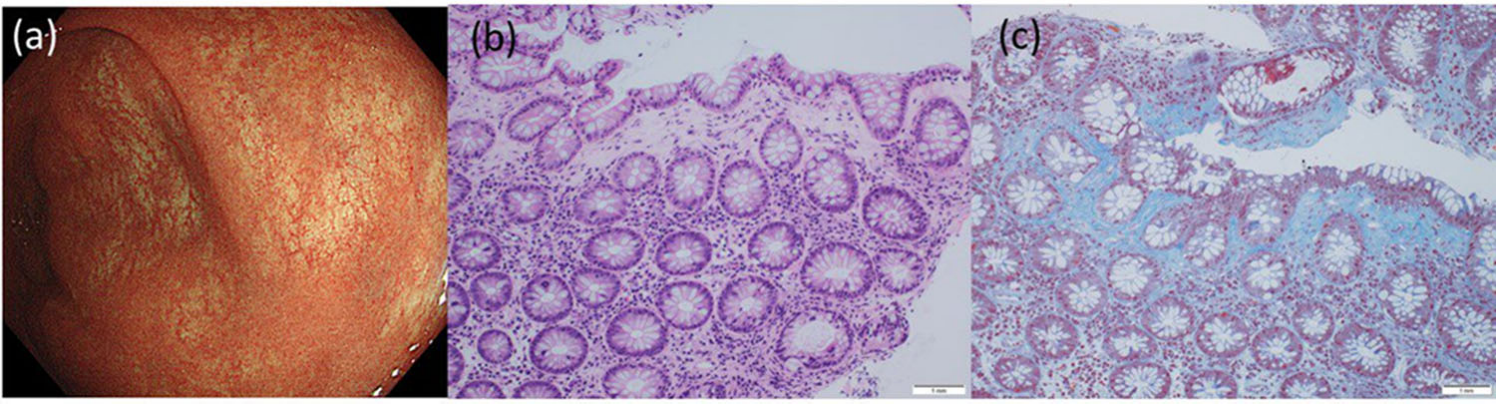
Case 1: A 78‐year‐old woman with non‐small cell lung cancer developed diarrhea 58 weeks after beginning treatment with nivolumab. (a) A colonoscopy revealed an indistinct vascular pattern. (b,c) Collagen bands were deposited in the biopsy specimen from the sigmoid colon

**FIGURE 2:**
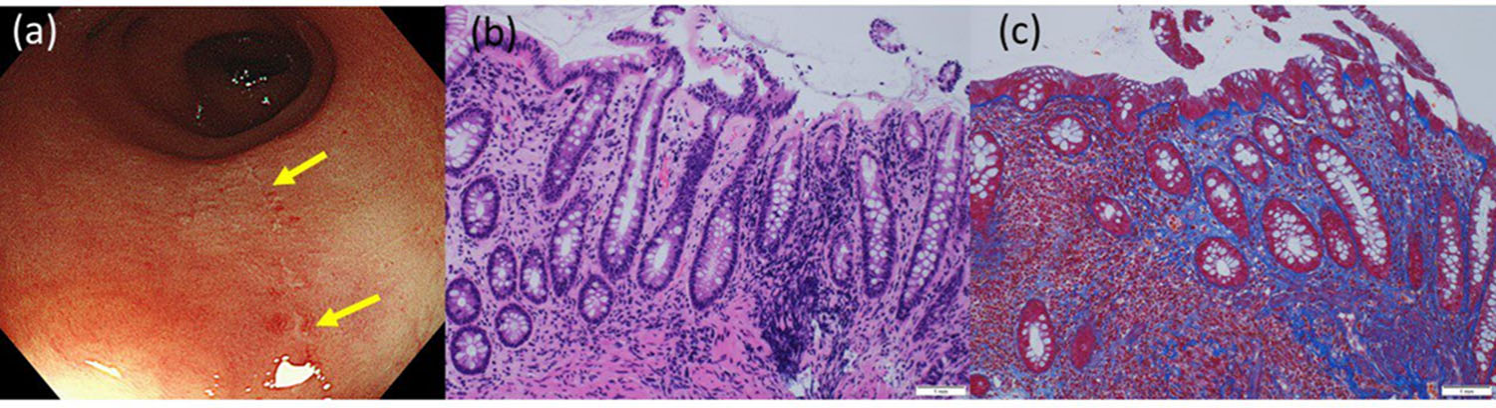
Case 5: An 83‐year‐old man with renal cell carcinoma developed diarrhea 3 weeks after beginning nivolumab and ipilimumab combination therapy. (a) A colonoscopy revealed a loss of vascularity and linear erosion (arrow). (b,c) Collagen bands were deposited in the sigmoid colon

**FIGURE 3:**
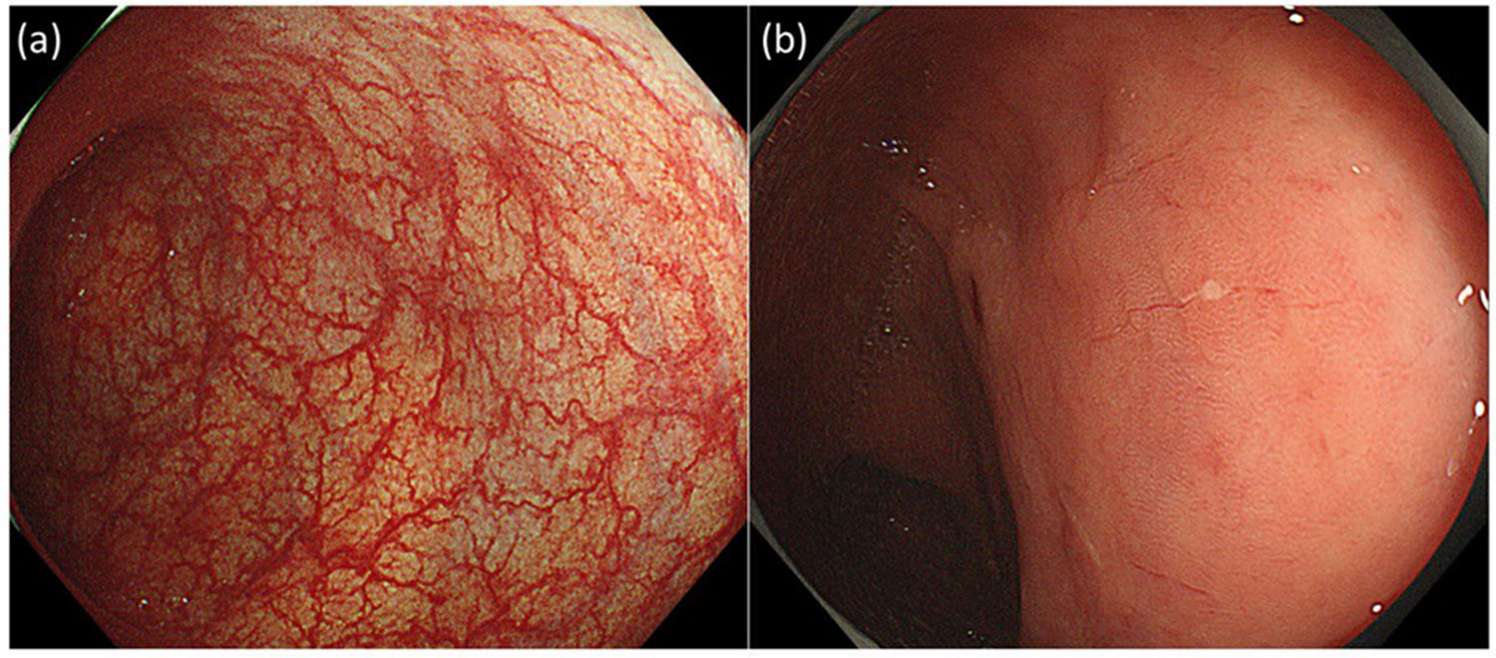
Case 8: An 81‐year‐old woman with malignant melanoma developed diarrhea 8 weeks after beginning treatment with nivolumab. (a) Normal mucosa was observed in the first colonoscopy, prior to the anti‐programmed cell death‐1 (anti‐PD‐1) treatment. (b) Loss of vascularity and erosion were seen in the second colonoscopy, after the anti‐PD‐1 treatment began

The endoscopic and histological findings for anti‐PD‐1‐related CC (n = 8), irAE colitis (n = 4), irAE ileitis (n = 1), and T‐CC (n = 9) are summarized in Table 2. Notably, all patients with anti‐PD‐1‐related CC showed indistinctness or loss of vascularity in the colorectal mucosa (Figure 1). Linear erosions similar to those of T‐CC were observed in three patients (Figure 2). None of the patients showed ulcers or hemorrhages in the colon or rectum. Interestingly, for patient #8, a colonoscopy prior to the induction of the anti‐PD‐1 treatment showed no mucosal changes. However, colonic mucosal changes, such as a loss of vascularity and erosion, were detected after anti‐PD‐1 administration, accompanied by persistent diarrhea (Figure 3).

**TABLE 2:** Summary of endoscopic and histologic findings

			Colonoscopy					Histology				
	Diagnosis	Case	Loss of vascularity	Erythema	Granularity	Erosion	Ulcer	Acute inflammation	Cryptitis	Crypt abscess	Apoptosis	Collagen band (μm)
ICI‐associated enterocolitis	Anti‐PD‐1‐related CC	1	±	−	−	−	−	−	−	−	−	30
		2	±	−	−	−	−	+	+	−	+	29
		3	+	−	−	−	−	−	−	−	−	12
		4	+	+	−	Linear	−	−	−	−	−	12
		5	+	+	−	−	−	+	−	−	+	13
		6	+	−	−	Linear	−	+	+	−	+	23
		7	±	−	−	−	−	+	+	−	+	25
		8	+	−	−	Linear	−	+	+	−	+	49
	irAE colitis	9	+	+	+	−	−	+	+	+	+	−
		10	+	+	+	+	+	+	+	+	+	−
		11	±	+	−	−	−	+	+	+	+	−
		12	+	+	+	+	+	+	+	+	+	−
	irAE ileitis	13	+	+	+	−	−	+	+	−	+	−
ICI‐independent traditional CC (T‐CC)		14	±	−	−	Linear	−	−	−	−	−	22
		15	±	+	−	−	−	−	−	−	−	41
		16	±	−	−	Linear	−	+	−	−	−	35
		17	±	+	−	−	−	−	−	−	−	70
		18	−	−	−	−	−	+	−	−	−	50
		19	−	−	−	−	−	−	−	−	−	40
		20	±	−	−	−	−	−	−	−	−	28
		21	−	−	−	−	−	−	−	−	−	30
		22	−	−	−	−	−	−	−	−	−	15

Abbreviations: anti‐PD‐1, anti‐programmed cell death‐1; CC, collagenous colitis; irAE, immune‐related adverse events.

**FIGURE 4:**
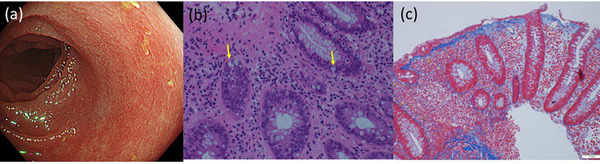
Case 4: An 83‐year‐old woman with renal cell carcinoma developed diarrhea 20 weeks after beginning pembrolizumab therapy. (a) A colonoscopy revealed a loss of vascularity and rough‐surfaced mucosa. (b) Apoptotic bodies were seen in the crypts (arrow). (c) Collagen bands were deposited in the descending colon

In the histopathological evaluations, collagen bands >10 μm were observed in the subepithelial layer for all the patients with anti‐PD‐1‐related CC and T‐CC. Collagen bands were visible in the sigmoid, transverse, and ascending colons of most of the patients with anti‐PD‐1‐related CC (Table 3, shaded area). Moreover, two patients had anti‐PD‐1‐related collagenous ileitis. In five of the anti‐PD‐1‐related CC cases, apoptotic bodies were observed in the crypts (Figure 4). Interestingly, most of the apoptotic bodies were found in the colorectal segments in which a collagen band was seen (Table 3, shaded area and “Apo”).

**TABLE 3:** Distribution of collagen band and presence of apoptosis

	Distribution of collagen band and presence of apoptosis						
Case	R	S	D	T	A	C	I
1	–	–	–	–	–	–	–
2	Apo	Apo	–	–	n/a	n/a	n/a
3	–	–	–	–	–	–	–
4	–	–	–	–	–	–	–
5	Apo	–	Apo	–	–	–	–
6	Apo	Apo	Apo	Apo	Apo	–	–
7	–	Apo	–	–	–	Apo	–
8	–	Apo	Apo	Apo	Post rt. hemicolectomy		–

Abbreviations: R, rectum; S, sigmoid colon; D, descending colon; T, transverse colon; A, ascending colon; C, cecum; I, ileum; n/a, not available.

Shaded areas signify the segments where a collagen band was seen.

Apoptotic body were detected in biopsy specimens from “Apo” segment.

**FIGURE 5:**
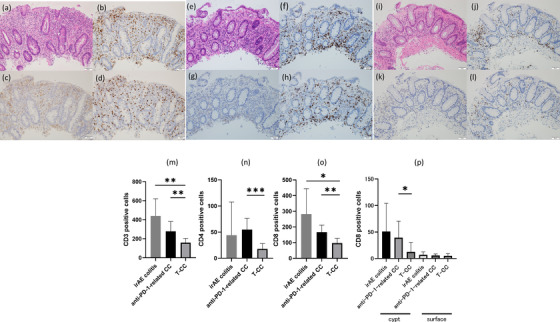
Immunohistochemical analysis. Immunohistochemical imaging of immune‐related adverse event (irAE) colitis using hematoxylin and eosin staining with representative staining patterns for CD3^+^, CD4^+^, and CD8^+^ cells (a, b, c, d). Immunohistochemical imaging of anti‐programmed cell death‐1 (anti‐PD‐1)‐related collagenous colitis (CC) using hematoxylin and eosin staining with representative staining patterns for CD3^+^, CD4^+^, and CD8^+^ cells (e, f, g, h). Immunohistochemical imaging of traditional collagenous colitis (T‐CC) using hematoxylin and eosin staining with representative staining patterns for CD3^+^, CD4^+^, and CD8^+^ cells (i, j, k, l). The number of infiltrated CD3^+^, CD4^+^, and CD8^+^ cells was significantly higher in the anti‐PD‐1‐related CC sample than in the T‐CC sample (m, n, o). Characteristically, the infiltration of CD8+ cells into the crypt, not surface epithelium, was significantly greater in the anti‐PD‐1‐related CC sample than in the T‐CC sample (p). (*p < 0.05, **p < 0.01, ***p <0.001)

Immunohistochemical analysis showed that most of the infiltrated inflammatory cells around the collagen band were CD3^+^ cells, suggesting that these inflammatory cells were primarily of T‐cell lineage. Moreover, the infiltration by CD8^+^ cells, which were localized in the subepithelial layer, was more compared to CD4^+^ cells. A similar tendency was observed in the histological assessments for the nine patients with T‐CC. However, the number of infiltrated CD3^+^, CD4^+^, and CD8^+^ cells was significantly higher in the lamina propria of the patients with anti‐PD‐1‐related CC than in those with T‐CC. Notably, the CD8^+^ cells showed greater infiltration into the crypts for the patients with anti‐PD‐1‐related CC than for those with T‐CC. The immunohistochemical findings of anti‐PD‐1‐related CC were similar to those of irAE colitis (Figure 5).

### Laboratory data and CT images

CRP levels were measured for all patients; fecal examination data, FC levels, and FIT results were not available for two patients (Table 4). The CRP and FC levels were significantly elevated in all the available samples. The FIT results were within the normal limit (<100 ng/ml) for two patients. Contrast‐enhanced CT was performed before the colonoscopy for six symptomatic patients. The CT images revealed low rates of intestinal wall thickening in three patients. Ascites and misty mesentery were not observed for any patient.

**TABLE 4:** Inflammatory biomarker levels and colorectal wall thickness on CT image

Case	CRP (mg/dl)	FC (μg/g)	FIT (ng/ml)	Colorectal wall thickness on CT image
1	1.9	n/a	n/a	n/a
2	0.663	3380	102	–
3	8.159	11500	57	Low
4	1.536	718	3369	n/a
5	6.155	n/a	n/a	low
6	2.21	5720	215	–
7	1.7	107	66	low
8	5.56	4244	2431	–

Abbreviations: CT, computed tomography; CRP, C‐reactive protein; FC, fecal calprotectin; FIT, fecal immunochemical test; n/a, not available.

### Clinical course

**FIGURE 6:**
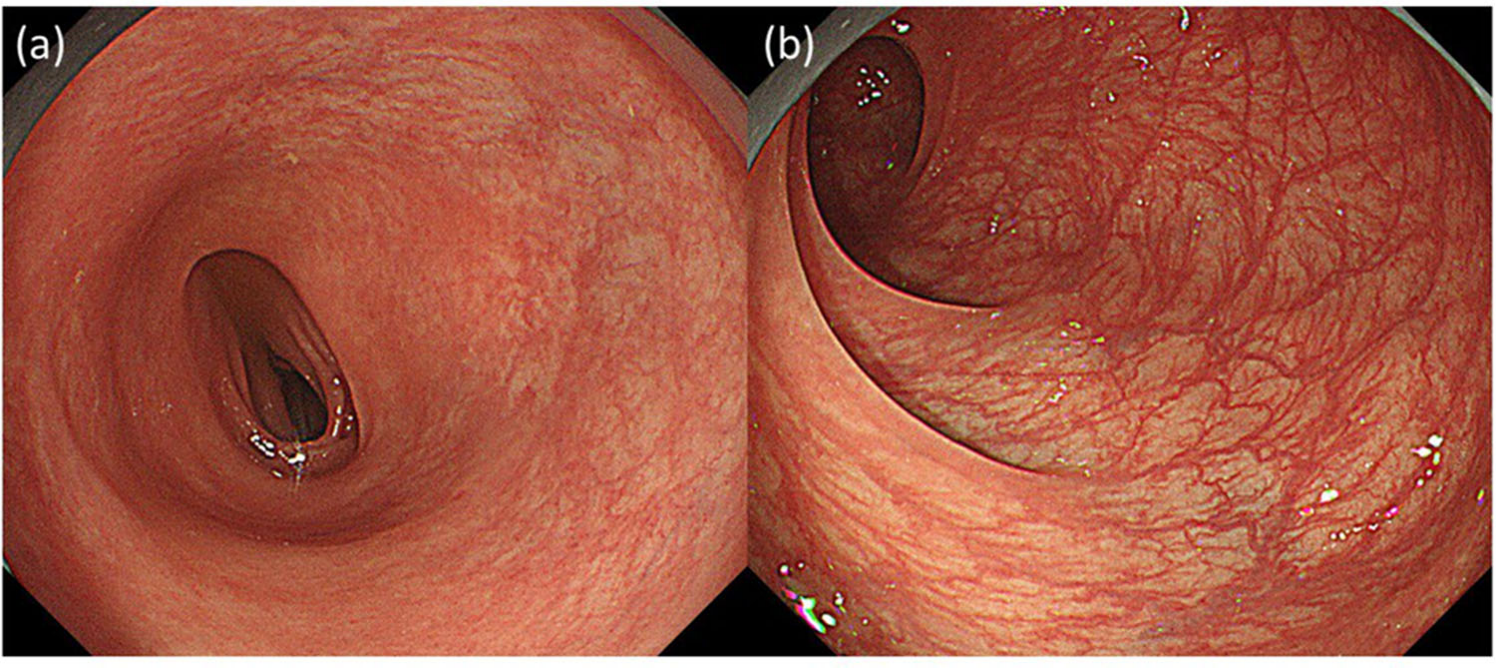
(a) Case 7: A 71‐year‐old man with non‐small cell lung cancer developed diarrhea 14 weeks after beginning pembrolizumab therapy. Indistinct vascularity was seen in the first colonoscopy image, while collagen deposits were detected histologically. (b) The second colonoscopy was performed after the proton pump inhibitor treatment was discontinued and the collagen band was no longer present in the biopsy specimen

The clinical course of the patients is summarized in Table 5. In the short term, the anti‐PD‐1/PD‐L1 treatment could be continued with suspension of the PPI for seven patients with mild disease. We performed colonoscopy and biopsies for one patient after the PPI was discontinued, post which the collagen band histologically disappeared (Figure 6). The patient with severe disease, who had to cease the ICI treatment, required intravenous prednisolone (PSL) administration; however, the patient died 6 months after anti‐PD‐1‐related CC diagnosis due to cancer progression. In the long‐term follow‐up, two patients were able to continue the ICI treatment for advanced cancer.

**TABLE 5:** Clinical course

		Cancer treatment after diagnosis of anti‐PD‐1 related CC				
Case	Gastric acid inhibitor	Short period	Long period	Outcome	Cause of death	Follow‐up period (week)
1	Discontinuation	ICI	Change to chemotherapy	Alive		161
2	Change to VPZ	ICI	Continuation of ICI	Alive		143
3	Change to VPZ	stop	Best supportive care	Death	Cancer progression	24
4	Discontinuation	ICI	Discontinuation of ICI due to irAE hepatitis	Death	Hepatic failure	3
5	Discontinuation	ICI	Discontinuation of ICI due to cancer progression	Death	Cancer progression	83
6	Change to VPZ	ICI	Continuation of ICI	Alive		54
7	Change to FA	ICI	Discontinuation of ICI due to irAE skin disorders	Alive		44
8	Change to FA	ICI	Discontinuation of ICI due to cancer progression	Death	Cancer progression	44

Abbreviations: VPZ, vonoprazan fumarate; FA, famotidine; anti‐PD‐1, anti‐programmed death‐1; CC, collagenous colitis; ICI, immune checkpoint inhibitor; irAE, immune‐related adverse event.

## DISCUSSION

To the best of our knowledge, this is the first report describing a series of patients who developed CC after being treated with ICIs. Our data show that CC was one of the most latent diseases in the patients presenting with diarrhea after anti‐PD‐1/PD‐L1 treatment. Although the patients’ endoscopic anti‐PD‐1‐related CC features could be obscure, with only mild colorectal mucosa inflammation, the pathological evaluations suggest that anti‐PD‐1‐related CC was characterized by dominant infiltration of CD8+ cells with localization into the crypts.

The etiology of CC remains controversial. Previous clinical studies have suggested certain risk factors for T‐CC, including genetic predisposition, immune disorders, gut microbiota, medications, and age.^16^, ^17^, ^18^, ^19^, ^20^ PPIs are well‐known inducers of CC.^21^ Conversely, the ICI‐related colitis risk factors include repeated ICI administration cycles, concomitant medications, a history of IBD, gut microbiota, and malignant melanoma.^22^, ^23^, ^24^

We reviewed 11 publications that described irAE colitis case series and minireviews.^2^, ^3^, ^8^, ^15^, ^25^, ^26^, ^27^, ^28^, ^29^, ^30^, ^31^ Detailed pathological investigations showed five cases of CC after an anti‐PD‐1/PD‐L1 treatment. We also reviewed three case reports describing this diagnosis.^9^, ^10^, ^11^ Although some data (such as endoscopically evident mucosal change, histological findings, concomitant drug use, or long‐term follow‐up outcomes) were unavailable, each of the three patients was reportedly treated for CC with budesonide or PSL. Thus, there remains no consensus regarding the importance of anti‐PD‐1/PD‐L1 antibody agents as risk factors for CC. In our study, eight patients with a recent medical history of PPIs developed CC after an additional treatment with anti‐PD‐1/PD‐L1 agents. Therefore, we argue that anti‐PD‐1/PD‐L1 antibody therapy could be a risk factor for CC in patients undergoing PPI therapy.

The endoscopic features of anti‐PD‐1‐related CC are characterized by mild inflammatory changes, even in patients suffering from severe diarrhea. In the present study, the most frequent colonoscopy finding was mucosal cloudiness, while erythema, granularity, and ulcer findings were relatively minor compared to those observed in typical irAE active colitis.

A histopathological anti‐PD‐1‐related CC diagnosis is based on the deposition of a subepithelial collagen band >10 μm. In the present study, the collagen band distribution showed that anti‐PD‐1‐related CC was not left‐side dominant, and had potential to extend to the ileum. Additionally, in the patients with anti‐PD‐1‐related CC, the crypts were predominantly infiltrated by CD8^+^ lymphocytes, rather than CD4^+^ lymphocytes. Göranzon et al. found a predominant accumulation of CD8^+^ lymphocytes in both the epithelium and lamina propria in patients with microscopic colitis, including those with CC and lymphocytic colitis.^32^ Another group found CD8^+^ T cells in the lamina propria and epithelium in patients with anti‐PD‐1‐induced colitis.^28^

Apoptotic bodies are often detected in the crypt in patients with PD‐1‐induced active colitis.^2^, ^15^, ^30^ In our study, in patients with anti‐PD‐1‐related CC, most of the apoptotic bodies were in the colorectal segments where collagen bands were observed. Anti‐PD‐1‐related CC was similar to T‐CC in terms of the presentation of the collagen band and predominant infiltration of CD8^+^ cells into the lamina propria, but differed in terms of the subepithelial cellularity and CD8^+^ cell infiltration into the crypt. In contrast, anti‐PD‐1‐related CC was similar to anti‐PD‐1‐induced active colitis in terms of the predominant CD8^+^ infiltration and increasing apoptotic body levels in the crypt, although the two diseases differed in terms of the collagen deposits. In other words, our patients with anti‐PD‐1‐related CC displayed features of both T‐CC and anti‐PD‐1‐induced active colitis.

Our findings indicate two clinical features of anti‐PD‐1‐related CC. The first is the lower incidence of intestinal wall thickening on CT images. The intestinal wall thickness, as measured on CT images, is a key factor to consider when assessing the severity and extent of active colitis due to irAE.^33^ Therefore, the absence of an increase in the intestinal wall thickness on CT images in patients with diarrhea after ICI treatment may be suggestive of anti‐PD‐1‐related CC, rather than active colitis due to irAE. To confirm the diagnosis of anti‐PD‐1‐related CC, a pathological examination following a colonoscopy and biopsy is essential.

The second key clinical feature of anti‐PD‐1‐related CC was the elevated FC levels. Calprotectin is a calcium‐binding protein primarily secreted by neutrophils and activated macrophages.^34^ FC is a clinical marker that shows high sensitivity and specificity for intestinal inflammation, especially in IBD; it is also useful as a biomarker for T‐CC.^35^ In the present study, the FC levels were increased in all patients with anti‐PD‐1‐related CC, including those who were FIT‐negative. However, because FC is not specific to CC, it cannot be used to distinguish anti‐PD‐1‐related CC from typical irAE colitis. Elevated FC levels indicate the presence of inflammation, which is found in both anti‐PD‐1‐related CC and irAE colitis. Therefore, an endoscopic examination is recommended for patients with elevated FC levels to distinguish anti‐PD‐1‐related CC from irAE colitis, even if the colorectal wall thickness is not increased on CT images. At the time of the initial diagnosis, the FC levels are not related to the diarrhea severity, endoscopic changes, number of infiltrated inflammatory cells, or collagen band thickness. However, after diagnosis of anti‐PD‐1‐related CC based on histological assessments, we believe that FC measurement might be useful as a non‐invasive biomarker of disease activity during follow‐up anti‐PD‐1‐related CC evaluations. This is noteworthy in relation to management strategies, given that all patients treated with ICI suffer from advanced cancer, and thus, unnecessary colonoscopy examinations should be avoided.

The National Comprehensive Cancer Network guidelines recommend that PSL, infliximab, or vedolizumab should be considered as treatment options for irAE colitis, in addition to symptomatic therapy and the suspension of ICI treatments.^33^ Alternatively, PSL, azathioprine, or mercaptopurine are considered acceptable for treating T‐CC, in conjunction with symptomatic therapy and the discontinuation of related drugs.^19^ Anti‐PD‐1‐related CC, which shows characteristics of both irAE colitis and T‐CC, requires the withdrawal of suspected drugs in mild cases and systemic administration of PSL in severe cases. Thus, consultations with clinical oncologists are essential for the treatment of anti‐PD‐1‐related CC and chemotherapy maintenance.

In this study, all patients who developed CC following anti‐PD‐1/PD‐L1 treatment had a recent history of PPI usage. Notably, patient #8 showed no endoscopic findings before the ICI treatment. However, when this patient experienced severe diarrhea after the ICI treatment, a second colonoscopy showed mild mucosal cloudiness and linear erosion. We assume that the PPI‐induced occult mucosal changes in the “first attack,” and the ICI elicited symptomatic diarrhea in the “second attack” contributed to complete the manifestation of anti‐PD‐1‐related CC. Hughes et al. reported that seven out of 13 patients with microscopic colitis after an ICI treatment had previously taken PPI medications.^36^ Another report also showed that PPI administration after ICI treatment resulted in CC.^37^ Thus, the mechanism underlying anti‐PD‐1‐related CC may involve immunological acceleration by anti‐PD‐1/PD‐L1 antibodies in combination with PPI‐induced intestinal changes, including intestinal dysbiosis or mucosal barrier weakness.

The limitations of this study include the single‐center model and small number of cases. Moreover, the target patients were limited to those who were referred to our department for gastroenterology investigations. Future studies should be performed with patients from multiple centers.

In conclusion, anti‐PD‐1/PD‐L1 treatments can cause anti‐PD‐1‐related CC in patients treated with PPIs. The features of anti‐PD‐1‐related CC include mild mucosal changes that are visible with endoscopy, regardless of elevated FC levels, and a lack of intestinal wall thickening on CT images. A histological assessment is essential for a diagnosis of anti‐PD‐1‐related CC. In this study, the histological features included predominant infiltration of CD8^+^ cells into the lamina propria and crypts, along with a subepithelial collagen band. The anti‐PD‐1‐related CC diagnosis allowed us to suspend the PPI administration while continuing chemotherapy. Therefore, we emphasize that a histological evaluation of biopsy specimens is crucial for patients with diarrhea after an ICI treatment, regardless of the severity of the endoscopic findings. We recommend that CC be considered as a differential diagnosis for patients with diarrhea during ICI treatments. Appropriate irAE assessments will contribute to maintaining each patient's quality of life.

## CONFLICT OF INTEREST

The authors have declared no conflict of interest.

## ETHICS STATEMENT

This study was approved by the ethics committee of Hirosaki University School of Medicine (No.2018‐1152) and conducted in accordance with the Declaration of Helsinki.

## FUNDING INFORMATION

None.
